# A missed opportunity: birth registration coverage is lagging behind Bacillus Calmette–Guérin (BCG) immunization coverage and maternal health services utilization in low- and lower middle-income countries

**DOI:** 10.1186/s41043-019-0183-3

**Published:** 2019-10-18

**Authors:** M. Hafizur Rahman, Amber Bickford Cox, Samuel L. Mills

**Affiliations:** 1 Johns Hopkins Bloomberg School of Public Health, Baltimore, MD 21205 USA; 2 World Bank Group, 1818 H Street, NW, Washington, DC 20433 USA

**Keywords:** Birth registration, Immunization, Maternal health service indicators

## Abstract

**Background:**

Civil registration and vital statistics (CRVS) systems lay the foundation for good governance by increasing the effectiveness and delivery of public services, providing vital statistics for the planning and monitoring of national development, and protecting fundamental human rights. Birth registration provides legal rights and facilitates access to essential public services such as health care and education. However, more than 110 low- and middle-income countries (LMICs) have deficient CRVS systems, and national birth registration rates continue to fall behind childhood immunization rates.

Using Demographic and Health Survey (DHS) and Multiple Indicator Cluster Survey (MICS) data in 72 LMICs, the objectives are to (a) explore the status of birth registration, routine childhood immunization, and maternal health services utilization; (b) analyze indicators of birth registration, routine childhood immunization, and maternal health services utilization; and (c) identify missed opportunities for strengthening birth registration systems in countries with strong childhood immunization and maternal health services by measuring the absolute differences between the birth registration rates and these childhood and maternal health service indicators.

**Methods:**

We constructed a database using DHS and MICS data from 2000 to 2017, containing information on birth registration, immunization coverage, and maternal health service indicators. Seventy-three countries including 34 low-income countries and 38 lower middle-income countries were included in this exploratory analysis.

**Results:**

Among the 14 countries with disparity between birth registration and BCG vaccination of more than 50%, nine were from sub-Saharan Africa (Tanzania, Uganda, Gambia, Mozambique, Djibouti, Eswatini, Zambia, Democratic Republic of Congo, Ghana), two were from South Asia (Bangladesh, Nepal), one from East Asia and the Pacific (Vanuatu) one from Latin America and the Caribbean (Bolivia), and one from Europe and Central Asia (Moldova). Countries with a 50% or above absolute difference between birth registration and antenatal care coverage include Democratic Republic of Congo, Gambia, Mozambique, Nepal, Tanzania, and Uganda, in low-income countries. Among lower middle-income countries, this includes Eswatini, Ghana, Moldova, Timor-Leste, Vanuatu, and Zambia. Countries with a 50% or above absolute difference between birth registration and facility delivery care coverage include Democratic Republic of Congo, Djibouti, Moldova, and Zambia.

**Conclusion:**

The gap between birth registration and immunization coverage in low- and lower middle-income countries suggests the potential for leveraging immunization programs to increase birth registration rates. Engaging health providers during the antenatal, delivery, and postpartum periods to increase birth registration may be a useful strategy in countries with access to skilled providers.

## Background

The United Nations (UN) defines civil registration as the “universal, continuous, permanent, and compulsory recording of vital events provided through decree or regulation in accordance with the legal requirements of each country” and defines vital statistics as “a collection of statistics on vital events in a lifetime of a person as well as relevant characteristics of the events themselves and of the person and persons concerned” [1]. Recording and documenting vital events in the population, including births, deaths, marriages, divorces, and adoptions, is a fundamental function of governments. Civil registration and vital statistics (CRVS) systems lay the foundation for good governance by increasing the effectiveness and efficiency of delivery of public services, providing vital statistics for the planning and monitoring of national development, and protecting fundamental human rights. Birth registration provides legal rights and facilitates access to essential public services such as health care and education. Birth certificates documenting the birth registration process provide proof of age, important evidence for strengthening gender empowerment issues including preventing child marriage, the right for women to own land, access credit, and vote. However, more than 110 low- and middle-income countries (LMICs) have deficient CRVS systems and are unable to effectively register and document birth, deaths, and marriages [2].

CRVS systems feature prominently in multiple UN Sustainable Development Goals (SDGs). Most notably, SDG target 16.9 stipulates that countries “by 2030, provide legal identity for all, including birth registration” [3] with the corresponding indicator 16.9.1 for monitoring the “proportion of children under 5 years of age whose births have been registered with a civil authority, by age” [4]. Increasing birth registration coverage in LMICs will contribute immensely to achieving SDG target 16.9. SDG 17.19 prioritizes initiatives supporting statistical capacity building efforts, specifically those to strengthen birth and death registration systems and to conduct a national census. In addition to the direct benefits of improving the quality and accuracy of vital statistics, improvements in CRVS can impact other SDGs by impacting poverty, education, and gender inequality [5].

Immunization tracking and record systems vary in coverage and quality. Conventional tracking systems include paper-based Expanded Program on Immunization (EPI) cards and regional clinic records, and though electronic health systems are uncommon at a national level, mHealth innovations are being used to provide software for national vaccine registries connecting vaccine records and mobile parental reminders. The World Health Organization has prioritized strengthening immunization systems in LMICs as part of a well-functioning health system in the Global Vaccine Action Plan [6]. The framework to deliver universal access to immunization to all by 2020 reflects the aligned vision of the key stakeholders of the Decade of Vaccines Collaboration, including the World Health Organization (WHO), the Bill & Melinda Gates Foundation, the United Nations Children’s Fund (UNICEF), and Gavi, the Vaccine Alliance, and with input from national governments, advocacy groups, funders, academia, and manufacturers [6, 7]. The plan includes elements to strengthen health information systems for childhood immunization records and CRVS systems, such as providing policy recommendations and implementation strategies. Unfortunately, in many countries, national birth registration rates continue to fall behind childhood immunization rates.

The birth registration process typically begins with a birth attendant completing and filing a birth notification form following the birth, whether the delivery is at home or at a facility. The BCG vaccination is also typically given by the birth attendant at the time of birth and documented in the birth record. The birth notification form is filed to the civil registration authority, and the parents are issued a birth certificate. Barriers to birth registration include a lack of social or institutional awareness of the benefits of birth registration, long distances to travel, costs or fees, and inefficiencies or inequalities in how CRVS systems are administered [8]. Currently, roughly 3 in 4 children live in sub-Saharan African countries where there are fees associated with birth registration, and in most cases, those fees reflect fines for late registration [8].

Routine childhood immunization and maternal health service programs may provide opportunities for collaboration through which birth registration systems could be improved. Using demographic and health survey (DHS) and multiple indicator cluster survey (MICS) data, the objectives of this article are to (a) explore the status of birth registration, routine childhood immunization, and maternal health service utilization in LMICs; (b) analyze indicators of birth registration, routine childhood immunization, and maternal health services in LMICs; and (c) identify missed opportunities for strengthening birth registration systems in countries with strong childhood immunization and maternal health services by measuring the absolute differences between the birth registration rates and these childhood and maternal health service indicators.

## Materials and methods

We constructed a database using DHS and MICS country data from 2000 to 2017, inclusive. Birth registration data were collected using DHS and MICS surveys for children younger than 5 years. In each type of survey, mothers were queried about whether each child’s birth had been registered administratively, whether they had a birth certificate, and whether they could produce the birth certificate.

The DHS and MICS surveys assessed immunization coverage rates by asking mothers of children aged 12 to 23 months about the current immunization status of their children at 12 months and asked whether vaccination cards were available. Current immunization status was surveyed for vaccines against tuberculosis, measles, diphtheria, pertussis, tetanus, and polio. For this analysis, we aligned with the DHS and MICS methods for vaccine coverage using any record of vaccination, either mother’s report or vaccination cards.

In addition to the birth registration and immunization coverage rates, data were extracted from DHS and MICS to analyze antenatal care (ANC) indicators, birth and delivery indicators, post-delivery care indicators for mother and newborn, wealth quintiles, and parental education and literacy indicators.

The dataset included information from LMICs and income levels were defined using the World Bank Country and Lending Groups 2018 update [9]. Low-income countries are those with a gross national income (GNI) per capita of $995 or less. Lower middle-income countries are those with a GNI per capita between $996 and $3895. If multiple surveys were available for a country during the 2000–2017 period, the most recent survey with the most comprehensive immunization and birth registration data available was selected.

We included countries based on the low- and lower middle-income countries’ classifications by the World Bank, excluding upper middle-income countries. We excluded countries with no recent DHS or MICS data available. Of the 72 countries included in the analysis, 69 had surveys providing immunization and birth registration data, and four countries reported one or the other but not both. DHS and MICS indicators in the database included birth registration for under 5 years, under 2 years, and under 1 year; immunization at 12 months in 12–23-month-old children by vaccine card or immunization record; and birth certificates in under-5-year-old and under-2-year-old children. We conducted exploratory and descriptive analyses for each type of indicator included in the database, with a focus on comparing under-1 birth registration data and 12-month-old immunization data. Stata statistical software version 14 (Stata Corp, College Station, TX, USA) was used to create the database and conduct the analyses.

## Results

### Birth registration and birth certificates of children younger than 5 years of age

**Fig. 1:**
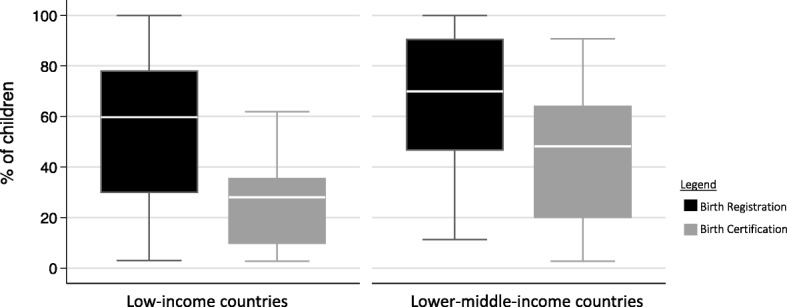
Birth registration and birth certification in children under 5 years in low-income and lower middle-income countries

All countries reporting both under-5 birth certificates and registration rates reported higher registration than certificates, with lower birth registration and certification rates in low-income countries compared to lower middle-income countries (Fig. 1).

Fewer than half of children younger than 5 years in each country had a birth certificate, with great variability in national birth certificate rates ranging from very low in Rwanda (2.7%), Zambia (4.1%), and Tanzania (7.7%) to over 99% in Egypt. Parents’ reports of birth registration were higher than reports of having a birth certificate in under-5 children (65.8% vs 31.1%) and in under-2 children (65.9% and 48.5%) (Table 1). Birth registration in under-1 children was 59.3%, but data was not available for birth certificates in under-1 children. Children in low-income countries were less likely than those in lower middle-income countries to have a birth registered or to have a birth certificate. Rates for children having a birth certificate and having their birth registered were similar regardless of sex in under-5 children. Rural children were less likely than urban children to have their birth registered or to have a birth certificate (Table 1).

**Table 1:** Summary median birth registration and birth certification in low-income and lower middle-income countries

Age of child (years)	Birth registration (%)			Birth certificates (%)	
	Under 5	Under 2	Under 1	Under 5	Under 2
All countries (*n* = 72)	*65.8*	65.9	59.3	*31.1*	48.5
Male	65.2			30.8	
Female	62.3			30.4	
Urban	76.4			38.1	
Rural	59.2			25.3	
Low-income countries (*n* = 34)	*58.3*	65.9	55.7	*28.0*	43.0
Male	58.0			26.2	
Female	58.6			24.2	
Urban	65.2			34.4	
Rural	55.0			23.0	
Lower middle-income countries (*n* = 38)	*72.4*	65.9	61.0	*50.9*	52.7
Male	68.1			51.5	
Female	66.7			50.3	
Urban	79.8			57.6	
Rural	60.5			46.6	

**Fig. 2:**
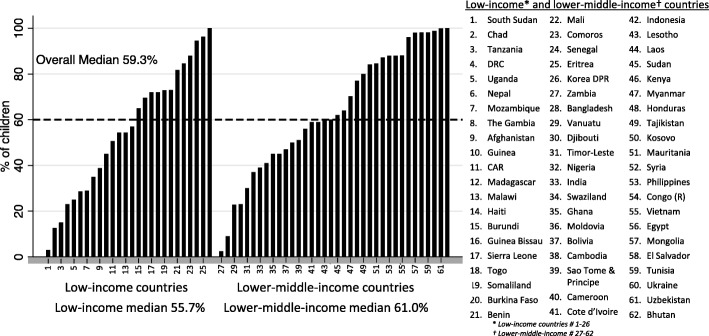
Birth registration in children under 1 year in low-income and lower middle-income countries

In both low-income and lower middle-income countries, the median birth registration occurs before the first birthday 60% of the time (Table 1, Fig. 2) while there is more variability in obtaining birth certificates, with the median lower middle-income country rate double that of the low-income (50.9% vs 28%) (Table 1).

**Fig. 3:**
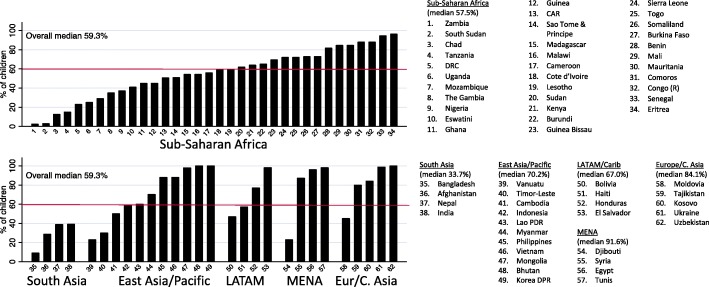
Birth registration in children under 1 year by the World Bank region

When birth registration of children under 1 year was stratified by the World Bank region, the median was lowest in South Asia (33.7%) and highest in the Middle East and North Africa (91.6%) and Europe and Central Asia (84.1%) (Fig. 3). When birth registration rates for children under 5 were analyzed according to sex and residence type (urban or rural), the rates did not differ significantly between boys and girls, although there was a higher proportion of birth registration for urban children than for rural children in East Asia and the Pacific, Latin America and the Caribbean, South Asia, and sub-Saharan Africa (data not shown).

### Immunization coverage at 12 months

**Fig. 4:**
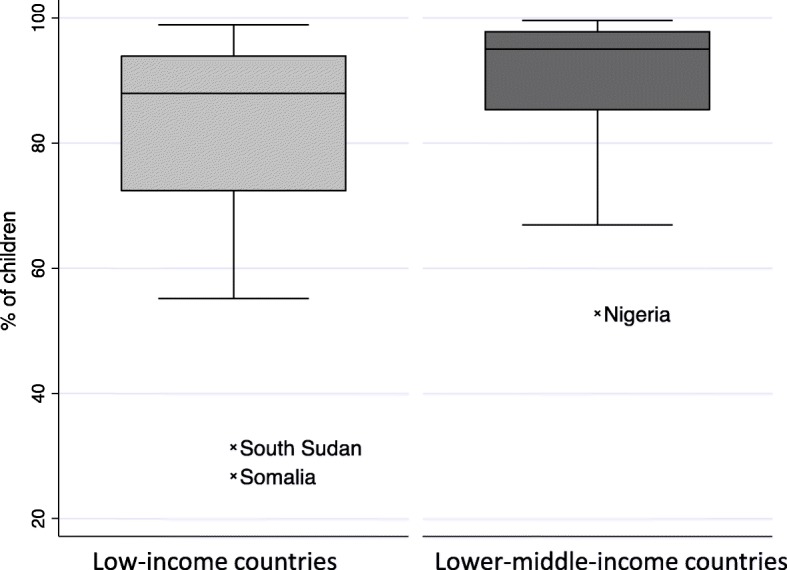
BCG immunization coverage at 12 months in children aged 12–23 months

**Fig. 5:**
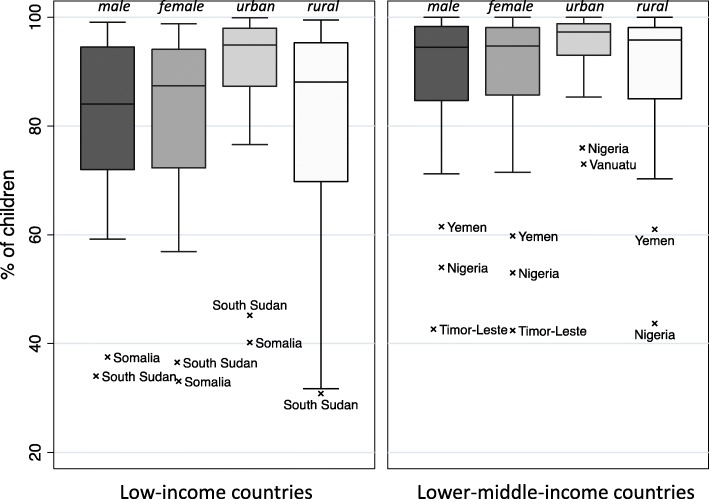
BCG immunization coverage at 12 months in children aged 12–23 months, by sex, and residence

In surveys of mothers of 12- to 23-month-old children, the median BCG immunization coverage for the 32 low-income countries was 88.2% but was higher at 95.9% for the 38 lower middle-income countries (Fig. 4). As expected, the coverage rate for children receiving the 9-month measle-containing vaccine was lower than that for BCG (at birth) and the three-dose series of DPT vaccines (typically at 6, 10, and 14 weeks) (not shown). BCG immunization coverage at 12 months was slightly lower in low-income countries than in lower middle-income countries (Fig. 5). Stratified analysis by the region showed that the median BCG immunization rate was highest in European and Central Asian regions (> 98%) and lowest in South Asian region (90%) (World Bank regions, data not shown).

In children aged 12 to 23 months, immunization coverage rates at 12 months were comparable in boys and girls, but rural children had lower immunization coverage rates than urban children (Fig. 5). Immunization coverage rates for four vaccines examined (BCG, DPT [diphtheria, pertussis, and tetanus], MCV [measles-containing vaccine], and polio) were higher for wealthier families and for children whose mothers had more education (data not shown).

### Absolute difference between birth registration and immunization

Tables 2 and 3 list the low- and lower middle-income countries, respectively, with the absolute difference between birth registration and BCG immunization rates, number of ANC visits, and delivery at a facility. Of the 14 countries with disparity between birth registration and BCG vaccination of more than 50%, nine were from sub-Saharan Africa (Tanzania 79.9%, Uganda 67.1%, Gambia 63.6%, Mozambique 61.3%, Djibouti 64.5%, Eswatini 56%, Zambia (98.6), DRC 61.0%, and Ghana 51.6%), two were from South Asia (Bangladesh 88.8% and Nepal 57.1%), one from East Asia and the Pacific (Vanuatu 56.3%), one from Latin America and the Caribbean (Bolivia 51.4%), and one from Europe and Central Asia (Moldova 52.8%).

**Table 2:** Birth registration, childhood Immunization, and maternal health service indicators in low-income countries (*N* = 34)

Low-income countries *N* = 34	Survey data source	World Bank region	Birth registration under 1 year	BCG 12 months	|BCG-BRR|**	≥ ANC1	|ANC > =1-BRR|+	Facility delivery*	|Facility delivery-BRR|++	BCG wealth inequality q5–q1	Birth registration wealth inequality q5–q1
Afghanistan	MICS 2010–2011	South Asia	38.8	61.3	22.5	73.4	34.6	32.9	5.9	24.9	26.7
Benin	MICS 2014	Sub-Saharan Africa	81.7	89.4	7.7	92.2	10.5	87	5.3	23.3	26.8
Burkina Faso	DHS 2010/MICS	Sub-Saharan Africa	73	96.2	23.2	95.7	22.7	71.6	1.4	15.5	22.4
Burundi	DHS 2010	Sub-Saharan Africa	65	98.8	33.8	98.9	33.9	64.5	.5	25.8	33.2
Central African Republic	MICS 2010	Sub-Saharan Africa	50.6	72.4	21.8	72.8	22.2	52.5	1.9	36.2	38.4
Chad	MICS 2010	Sub-Saharan Africa	12.7	55.2	42.5	25	12.3	23.6	10.9	21.5	51
Comoros	MICS 2000	Sub-Saharan Africa	88	84.6	3.4			77.5	10.5	35.1	8.8
DRC	MICS 2010	Sub-Saharan Africa	23	84	61	89.5	66.5	80.4	57.4	25.5	2
Eritrea	2002 DHS	Sub-Saharan Africa	96.3	89.3	7	72.1		28.3	68	20.1	
Ethiopia	DHS 2005	Sub-Saharan Africa		65.2		33.7		11		1.7	15
Gambia	MICS 2010	Sub-Saharan Africa	35	98.6	63.6	98.1	63.1	63.3	28.3		6.3
Guinea	MICS 2016	Sub-Saharan Africa	45	81.6	36.6	74.6	29.6	57.2	12.2	5.2	35
Guinea-Bissau	MICS 2014	Sub-Saharan Africa	69.6	90.5	20.9	90.9	21.3	44	25.6	9.2	18
Haiti	DHS 2012	Latin America and the Caribbean	57	80.6	23.6	89.6	32.6	38.1	18.9	6.4	20.5
Korea DPR	MICS 2009	East Asia and Pacific	100			98.7	1.3	94.7	5.3		
Liberia	DHS 2007	Sub-Saharan Africa		93.3		96.2		59.4		27.1	14.9
Madagascar	MICS 2012	Sub-Saharan Africa	54.3	63.5	9.2	77.1	22.8	25.3	29	28.8	31.5
Malawi	MICS 2013–2014	Sub-Saharan Africa	54.3	96.3	42	96.8	42.5	88.9	34.6	1.1	− 2.8
Mali	MICS 2015	Sub-Saharan Africa	84.6	71.9	12.7	75.5	9.1	64.5	20.1	34.3	25.7
Mozambique	DHS 2011	Sub-Saharan Africa	29	90.3	61.3	90	61.0	57.2	28.2	21.9	17.9
Nepal	MICS 2014	South Asia	28.6	85.7	57.1	85.6	57.0	55.2	26.6	1.1	16.5
Niger	MICS/DHS 2006	Sub-Saharan Africa		82.6		66		33		31.8	39.4
Rwanda	DHS 2010	Sub-Saharan Africa		98.9		98.1		90.7		2.1	20.9
Senegal	MICS/DHS 2010–2011	Sub-Saharan Africa	94.5	93.7	.8	91.9		77.9	16.6	1.4	44
Sierra Leone	MICS 2017	Sub-Saharan Africa	72	93.9	21.9	33	39.0	55	17	1.1	14
Somalia	MICS 2006	Sub-Saharan Africa	72.8	26.8	46	41.3	31.5	30.6	42.2	14.2	6
South Sudan	MICS 2010	Sub-Saharan Africa	3	31.4	28.4	41.4	38.4	11.5	8.5	38.1	35.3
Syria	MICS 2006	Middle East and North Africa	87.2	89.6	2.4	85.3	1.9	70.4	16.8	10.4	34
Tajikistan	DHS 2012	Europe and Central Asia	80	98.3	18.3	79.9	0.1	78	2	2.6	4
Tanzania	DHS 2010	Sub-Saharan Africa	15	94.9	79.9	97.7	82.7	50.2	35.2	.5	51.4
Togo	MICS 2010	Sub-Saharan Africa	72	95.1	23.1	86.7	14.7	74.9	2.9		38
Uganda	DHS 2011	Sub-Saharan Africa	25	92.1	67.1	94.3	69.3	59.1	34.1	13.1	17
Yemen	DHS 2013	Middle East and North Africa		66.9		60.9		31		48.3	39
Zimbabwe	2010–2011 DHS	Sub-Saharan Africa		86.6		87.6		64.1		20.6	39.5

*Delivery within the last 3 years

**Mali, Senegal—the difference is negative

+Eritrea, Korea DPR, Senegal, Sierra Leone, Somalia, Syria, Tajikistan—the difference is negative

++Afghanistan, Burkina Faso, Burundi, Central African Republic, Comoros, Eritrea, Guinea-Bissau, Haiti, Korea DPR, Madagascar, Mali, Senegal, Sierra Leone, Somalia, Syria, Tajikistan—the difference is negative

**Table 3:** Birth registration, childhood immunization, and maternal health service indicators in lower middle-income countries (*N* = 38)

Lower middle-income countries *N* = 38	Survey data source	World Bank region	Birth registration under 1 year	BCG 12 months	|BCG-BRR|**	≥ ANC1	|ANC > =1-BRR|+	Facility delivery*	|Facility delivery-BRR|++	BCG wealth inequality q5–q1	Birth registration wealth inequality q5–q1
Bangladesh	MICS 2012–2013	South Asia	9	97.8	88.8	47.7	38.7	16	7	3.3	17
Bhutan	2010 MICS	East Asia and Pacific	100			97.4	2.6	63.1	36.9		0
Bolivia	DHS 2008	Latin America and the Caribbean	47	98.4	51.4	90.1	43.1	69.3	22.3	20.7	22.4
Cambodia	DHS 2010	East Asia and Pacific	50	95.9	45.9	90.5	40.5	87	37	.8	30
Cameroon	MICS 2014	Sub-Saharan Africa	56	91.2	35.2	82.4	26.4	61.3	5.3	18.2	60.8
Cote d'Ivoire	MICS 2016	Sub-Saharan Africa	59	78.3	19.3	93.3	34.3	69.8	10.8	31.7	46
Djibouti	MICS 2006	Middle East and North Africa	23	87.5	64.5			87.4	64.4		
Egypt	MICS 2013–2014	Middle East and North Africa	96	91.1	5.0	90.7	5.3	82.1	13.9		1
El Salvador	MICS 2014	Latin America and the Caribbean	98.1	97.7	.4	96.5	1.6	97.5	.6	.2	
Eswatini	2014 MICS	Sub-Saharan Africa	41	97	56	94.9	53.9	75.1	34.1		34
Ghana	MICS 2011	Sub-Saharan Africa	45	96.6	51.6	96.7	51.7	75.3	30.3	5.2	35
Honduras	DHS 2011–2012	Latin America and the Caribbean	77	98.4	21.4	96.6	19.6	84.7	7.7	23.4	3.6
India	NFHS 2005–2006	South Asia	39	75.6	36.6	76.3	37.3	40.8	1.8	31.4	48.5
Indonesia	DHS 2012	East Asia and Pacific	59	88.6	29.6	96.3	37.3	66.8	7.8	18.9	47.4
Kenya	2014 DHS	Sub-Saharan Africa	64	95.9	31.9	95.7	31.7	61.2	2.9	5.6	36.6
Kosovo	MICS 2013–2014	Europe and Central Asia	84.1	98.7	14.6	97.8	13.7	99	14.9	1.6	12.1
Kyrgyz Republic	2014 MICS	Europe and Central Asia		99.6		98.4		98.3		1.2	− .1
Lao PDR	MICS 2017	East Asia and Pacific	60.1	81.5	21.4	54.2	5.9	64.5	4.4	20.9	26.9
Lesotho	2009 DHS	Sub-Saharan Africa	59.6	97.6	38	89.3	29.7	79	19.4	35.5	28.6
Mauritania	MICS 2015	Sub-Saharan Africa	84.6	83.2	1.4	80.6	4.0	69.3	15.3	13.9	51
Moldova	MICS 2012	Europe and Central Asia	45	97.8	52.8	98.8	53.8	98.9	53.9	− 4.9	1
Mongolia	MICS 2013	East Asia and Pacific	98	97.9	.1	98.7	0.7	98.5	.5	1.2	.6
Morocco	DHS 2003–2004	Middle East and North Africa		97.8		38.3		63.1		24.6	
Myanmar	MICS 2009–2010	East Asia and Pacific	70.2	97.2	27	93.1	22.9	36.2	34	.3	45.5
Nicaragua	DHS 2001	Latin America and the Caribbean		94.8		85.3		69.3		23.8	
Nigeria	MICS 2016–2017	Sub-Saharan Africa	37	52.8	15.8	65.8	28.8	37.5	.5	63.9	64
Pakistan	DHS 2006–2007	South Asia		83.2		65.2		52.7		.8	66.4
Philippines	2010 Census	East Asia and Pacific	88	94.8	6.8	95.9	7.9	66.2	21.8	1.3	
R-Congo	DHS 2011–2012	Sub-Saharan Africa	88	93.3	5.3	92.2	4.2	91.8	3.9	10.7	19
Sao Tome and Principe	2014 MICS	Sub-Saharan Africa	51	95.2	44.2	97.5	46.5	91	40	− 2.2	11.1
Sudan	MICS 2014	Sub-Saharan Africa	62	78.5	16.5	74.3	12.3	27.7	34.3	26.6	60.9
Timor-Leste	DHS 2009–2010	East Asia and Pacific	30	76.6	46.6	86.7	56.7	23.8	6.2		51.4
Tunisia	MICS 2011–2012	Middle East and North Africa	98.1	98.1	0	97	1.1	98.5	.4	.6	38
Ukraine	MICS 2012	Europe and Central Asia	98.8	94.5	4.3	98.6	0.2	99.6	.8	4.0	17
Uzbekistan	2006 MICS	Europe and Central Asia	99.9	99.2	.7	99	0.9	97.3	2.6	0	− .9
Vanuatu	MICS 2007–2008	East Asia and Pacific	22.8	79.1	56.3	98.1	75.3	79.8	57	− 12.8	.1
Vietnam	2014 MICS	East Asia and Pacific	88.1	98	9.9	87.1	1.0	78.5	9.6	10.4	27.6
Zambia	DHS 2013–2014	Sub-Saharan Africa	2.5	94.1	91.6	98.2	95.7	67.4	64.9	5.1	9.3

*Delivery within the last 3 years

**Egypt, El Salvador, Mongolia, Ukraine, Uzbekistan—the difference is negative

+Bhutan, Egypt, El Salvador, Lao PDR, Mauritania, Tunisia, Ukraine, Uzbekistan, Vietnam—the difference is negative

++Bhutan, Egypt, El Salvador, Kenya, Mauritania, Myanmar, Philippines, Sudan, Timor-Leste, Uzbekistan, Vietnam—the difference is negative

### Antenatal care coverage

In low- and lower middle-income countries, a median of 90.3% of women had at least one or more ANC visits (93.3% in low- and 86.7% in lower middle-income countries), and the majority of women saw a provider by 4 months for their first visit. Women in lower middle-income countries had more ANC visits on average than those in low-income countries (data not shown).

### Absolute difference between birth registration and ANC coverage

Birth registration rates and rates of maternal ANC visits are shown in Tables 2 and 3. Countries with a 50% or above absolute difference between birth registration and ANC coverage include DRC (66.5%), Gambia (63.1%), Mozambique (61.0%), Nepal (57.0%), Tanzania (82.7%), and Uganda (69.3%) in low-income countries. Among lower middle-income countries, this includes Eswatini (53.9%), Ghana (51.7%), Moldova (53.8%), Timor-Leste (56.7%), Vanuatu (75.3%), and Zambia (95.7%).

### Facility delivery care coverage

Women in low- and lower middle-income countries who had at least one ANC visit within the last 2 to 3 years were more likely to have seen a skilled provider (including a nurse or doctor) for their delivery care rather than an unskilled provider (traditional birth attendant or community health worker). Three countries (South Sudan, Niger, and Nigeria) had the highest rates of women who delivered without a skilled attendant at birth (data not shown). In all countries, more women delivered in public than private facilities.

### Absolute difference between birth registration and facility delivery care coverage

Tables 2 and 3 show birth registration and facility delivery rates and the absolute difference between these rates. Several countries maintain high rates for birth registration, and facility deliveries, including Uzbekistan. In DRC and Vanuatu, the rates of facility deliveries are 79% and 80%, respectively, while their birth registration rates are less than 25%. Ethiopia and Somalia both report single-digit birth registration rates. In all LMICs included, the median for facility deliveries is 66.5%. The median in lower middle-income countries (72.5%) is higher than in low-income countries (58.2%). Countries with a 50% or above absolute difference between birth registration and facility delivery care coverage include DRC (57.4%), Djibouti (64.4%), Moldova (53.9%), and Zambia (64.9%).

### Wealth inequality and differences in birth registration and immunization

**Fig. 6:**
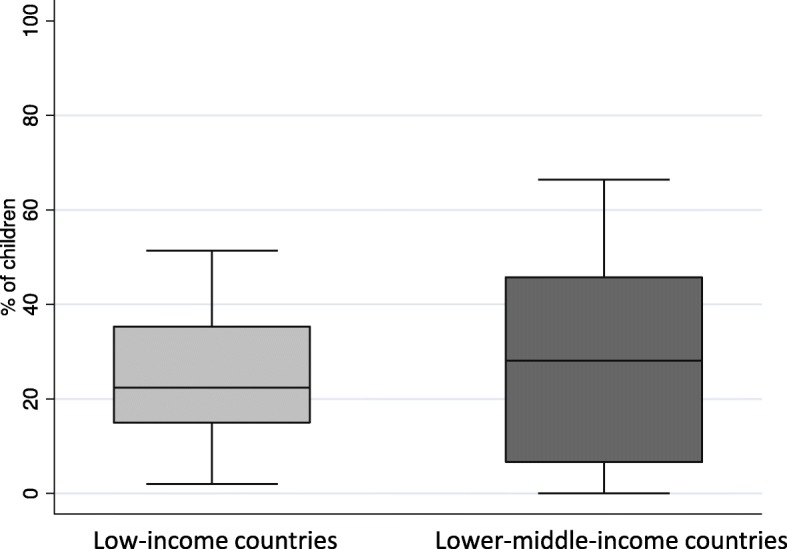
Birth registration and wealth inequality in children under 5 in low-income and lower middle-income countries

Plotting the differences of birth registration between the wealthiest quintile and the poorest quintile in children under 5 showed a more significant disparity in lower middle-income countries between the richest and the poorest. Figure 6 reveals the spread of differences in birth registration of the richest wealth index quintile to the poorest wealth index quintile in low- and lower middle-income countries. In half of the low-income countries, the difference in birth registration between the richest and the poorest is greater than 24.1% (median) whereas the median for the lower middle-income countries is 27.6%.

**Fig. 7:**
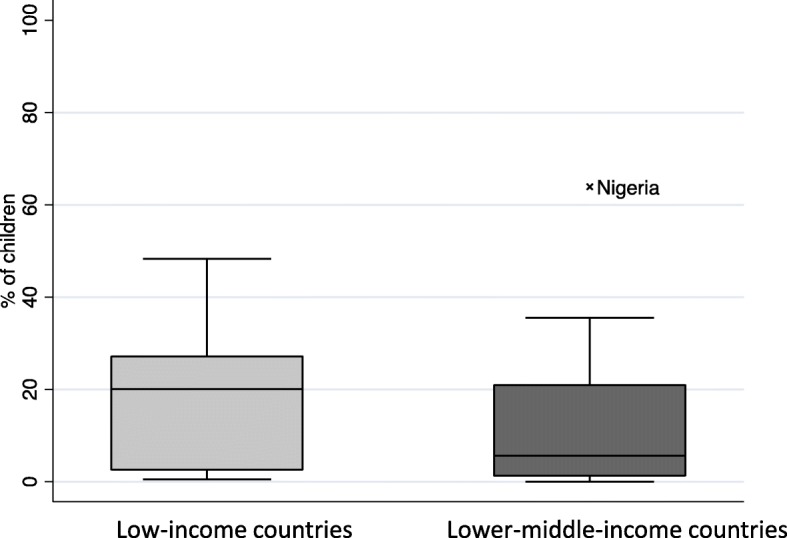
Bacillus Calmette–Guérin immunization coverage and wealth inequality in children under 12 months in low- and lower middle-income countries

In contrast, Fig. 7 reveals the spread of differences in reported immunization with the first dose of BCG in children in the richest wealth index quintile compared to the poorest wealth index quintile in LMICS. In half of the low-income countries, the difference between wealthiest and poorest BCG coverage is greater than 20.1%, whereas in lower middle-income countries, the median difference in BCG coverage between the wealthiest and poorest is much lower (median 5%) with an outlier country Nigeria where the difference is 63%.

## Discussion

This study significantly contributes to global efforts to understand the status of maternal and child health service indicators and birth registration in low- and lower middle-income countries. We found variability in birth registration rates between low-income and lower middle-income countries. Birth registration in children younger than 12 months of the 72 countries included in this analysis ranged from nearly 0 to 100% (median 59.3%). Regional birth registration medians ranged from the lowest levels in South Asia (33.7%) and sub-Saharan Africa (57.5%), to the highest levels in the Middle East and North Africa (91.6%) and in Europe and Central Asia (84.1%).

It was also found that reported rates of issuance of birth certificates in low- and lower middle-income countries were lower than reported birth registration rates, and the median under-5 birth certification rate for all countries is 31.1%. Having a birth certificate provides certain privileges and protections to children and parents. For example, health care services are free for children younger than 6 years in Vietnam but only if they have an insurance card, which is obtained only with a birth certificate. Some countries such as India, Kenya, and Sierra Leone provide children with universal access to health care without a birth certificate [10].

Similarly, education administrations may require proof of birth registration for school enrollment. In Vietnam, a child needs a birth certificate to be enrolled in school [10]. In Bangladesh, an individual needs to show a birth certificate to enroll in school or to obtain a passport [11]. In Brazil, one must show a birth certificate to obtain citizenship, to graduate from school, and to apply for social security [11]. In South Africa, citizens need a birth certificate to receive child welfare grants [11].

National immunization coverage rates are higher than birth registration rates in both low- and lower middle-income countries, with median BCG coverage of 89.3% and 95.2% in low-income countries and lower middle-income countries, respectively. Electronic birth registration, immunization record systems, and maternal and infant health tracking are being explored and implemented in different countries through clinic-based and community outreach programs [12].

mHealth and digital health innovations are optimizing the use of electronic technologies to accelerate the process of bringing paper-based systems online with cell phone and digital platforms [13]. These systems range from mobile and short message service (SMS)-based registration to web server-based online systems. Open MRS, Open SRP, and DHIS2 are software programs developed for registering health-related information in developing countries so that they can transition from traditional paper-based systems. Health care workers can operate these programs, such as Open SRP, to register their clients using an android application that the health care providers can run on a tablet or smartphone [14]. This registration is then used to facilitate the provision of reproductive, maternal, newborn, and child health care services [14].

In Nigeria, Open MRS has been used since 2009 to gather data about family planning, antenatal appointments, deliveries, child health, and immunizations [15]. DHIS2 has been used in Ghana since 2012 to register monthly data and information on people in hospitals to help increase the precision of statistics on morbidity and mortality [7]. In Tanzania, DHIS2 has been used to predict immediate outbreaks by collecting data from various health services [7]. Tanzanian registrars are also using mHealth technology to transmit birth registration information to a central system for data collection and storage [2, 16]. In Uganda, the National Identification and Registration Authority reviews data that collectors send through SMS [17]. Once the registrar verifies the data, a birth certificate is produced for the family. In Uruguay, the birth registration process is web-based, and newborns receive their birth certificates before they leave the hospital [2].

In Cambodia, a periodic campaign strategy to register births using mobile or mHealth technology has been used since 2004 and contributed to reaching 90% birth registration coverage in 2015 [18]. Another mobile registration system, Orange Mobile Birth Registration Solution in Senegal, provides village chiefs with mobile phones to directly notify the Senegal state register about births and deaths in the village. The system uses a Java applet that provides better customization options and customer experience than SMS-based notification systems [2].

The Millennium Village Project initiated in Kenya enabled community health workers to use SMS technology to register infants and monitor the health of children younger than 5 years. This strategy provided an opportunity to create and maintain a child registry and to monitor risk factors related to child mortality [19]. In Bangladesh, a similar program began providing digital tablets to health care workers to collect client health-related data. Collecting data digitally can be less time-consuming and more accurate than using a paper-based system [20]. Academic researchers and government planners have supported a successful strategy used in Bangladesh to improve vaccination coverage by linking mobile interventions with a web system called mTika [21]. In this system, after a pregnant woman is enrolled, health workers provided timely SMS reminders about her children’s vaccinations. These methods have improved vaccination rates in remote areas [21]. In Vietnam, PATH has developed and implemented a digital registry for tracking individual client immunization history and local vaccine stocking in collaboration with the National Expanded Program on Immunization (NEPI) that was less time-consuming for local staff, permitted greater geographic coverage, and increased national vaccination rates [22]. Most of these platforms can also be used to improve birth registration.

Just as with immunization coverage, ANC and delivery care coverage far surpasses that of birth registration in low- and lower middle-income countries. Thus, it may work to use the delivery of maternal and child services to boost birth registration coverage in low- and lower middle-income countries. For instance, pregnant women could be educated on the benefits of birth registration and encouraged to register their births. For facility deliveries, birth attendants could fill out the birth notification form to initiate the birth registration process. For home births, immunization personnel could check during outreach services or at immunization clinics to see whether births are registered and to complete birth notification forms or refer mothers to the appropriate personnel for birth registration. Birth notifications could also be incorporated into any of the electronic health information systems noted earlier to facilitate the birth registration process.

There are limitations of using survey research. It is possible that we have included outdated statistics for certain countries or that more recent reports would be representative of progress in birth registration, such as in Ethiopia where birth registration has only formally started since 2016. In the case of Ethiopia, we used immunization and maternal health services data but under-1 birth registration data were not yet available from DHS. However, the DHS and MICS have provided useful data for this analysis. Future surveys should expand birth certificate and registration data among under-1 children. Further research is also needed to understand the impact of wealth inequality and the differences in birth registration and immunization coverage in LMICs.

## Conclusion

Studies indicate that better CRVS systems are associated with better health and wealth outcomes, justifying the financial and technological investments needed to strengthen CRVS systems [23]. Birth registration provides one of the most basic forms of protection of the identity of children, particularly vulnerable children born during emergency situations [24]. Establishing systems for the accurate and timely recording of vital statistics and cause-of-death data also helps meet international development objectives [25].

The gap between birth registration and immunization coverage in low- and lower middle-income countries that this study has demonstrated suggests the potential for leveraging immunization programs to increase birth registration rates [26]. Study findings warrant special attention to the most vulnerable individuals living in rural areas and regions where birth registration is low. Engaging health providers during the antenatal, delivery, and postpartum periods to increase birth registration may be a useful strategy in countries with access to skilled providers.

## Data Availability

Not applicable

## Abbreviations

ANC: Antenatal care
BCG: Bacillus Calmette–Guérin
CRVS: Civil Registration and Vital Statistics
DHS: Demographic and Health Survey
DPT: Diphtheria, Pertussis, Tetanus
DRC: Democratic Republic of Congo
EPI: Expanded Program on Immunization
GNI: Gross National Income
LMICs: Low- and middle-income countries
MICS: Multiple Indicator Cluster Survey
NEPI: National Expanded Program on Immunization
SDG: Sustainable Development Goals
SMS: Short message service
UN: United Nations
UNICEF: United Nations Children’s Fund
WHO: World Health Organization
